# Should children with a ventriculo-peritoneal shunt play rugby: a survey of UK and Ireland paediatric neurosurgeons

**DOI:** 10.1007/s00381-026-07310-z

**Published:** 2026-05-13

**Authors:** Isabel Key, David Lowes, Paul Leach

**Affiliations:** 1 Cardiff University School of Medicine, Cardiff, UK; 2 Department of Paediatric Neurosurgery, University Hospital of Wales and Noah’s Ark Children’s Hospital for Wales, Cardiff, UK

**Keywords:** Paediatric neurosurgery, Ventriculoperitoneal shunt, Hydrocephalus, Contact sports, Rugby, Return to play, Sport participation

## Abstract

**Background:**

Ventriculoperitoneal shunt (VP) insertion is a common intervention in paediatric neurosurgery, and as outcomes for children with hydrocephalus improve, clinicians are increasingly asked to provide advice beyond the immediate postoperative period. Participation in sport is an important component of childhood development, yet there is limited evidence or consensus guidance to inform recommendations regarding contact sports for children with VP shunts. As a result, clinical advice is often based on individual experience rather that robust data.

**Methods:**

A structured electronic questionnaire was distributed to members of the British Paediatric Neurosurgery Group (BPNG). Participants were asked about their experience of rugby related VP shunt complications, the levels of rugby participation they would permit at different ages and whether they would recommend the use of protective head gear.

**Results:**

Overall response rate of 42.8% was achieved (30 of 70 respondents). Only one respondent (3%) reported direct experience of rugby related shunt complication. All respondents would permit a 7-year-old with a VP shunt to participate in touch rugby. 80% would allow a 14-year-old to play contact rugby, and 77% would permit an 18-year-old to participate in professional or elite rugby. The use of a scrum cap was recommended by 77% of respondents.

**Conclusions:**

Most paediatric neurosurgeons support return to rugby for children with VP shunts, commonly recommending protective headgear. These findings support the importance of providing informed, individualised guidance for resuming play.

## Introduction

Ventriculoperitoneal (VP) shunt insertion is a commonly performed procedure in paediatric neurosurgical practise. As survival and long-term outcomes for children with hydrocephalus continue to improve [1], paediatric neurosurgeons are increasingly asked to provide guidance beyond the postoperative period. Undoubtedly, participation in sport represents an important component of physical, psychological, and social development in childhood [2]. Consequently, patients and their families commonly seek advice regarding the appropriateness of different levels and types of sporting activities for children with VP shunts. Despite the frequency of these discussions, there is a notable paucity of evidence-based guidance or consensus recommendations to inform neurosurgeons advice. Resulting in clinical decision-making being based off individual experience rather than robust data. As a result, in this study we aimed to explore paediatric neurosurgeons’ perspectives on return to participation in the contact sport, rugby, following a VP shunt insertion. To address this, we conducted a nationwide survey of paediatric neurosurgeons in the UK and Ireland, to identify the current advice and recommendations regarding return to rugby for children with VP shunts (see Appendix). We also reviewed the other evidence of contacts sports and VP shunts.

## Methods

A questionnaire was emailed out to the current membership of the British Paediatric Neurosurgery Group (BPNG) after ratification by the Society of British Neurological Surgeons academic committee. A total of 70 members were contacted. The electronic questionnaire was distributed by email on three occasions between October 2025 and December 2025.

The questionnaire compromised five dichotomous questions designed to assess clinical experience and decision-making regarding levels of rugby participation in children with VP shunts (see Appendix).

The questionnaire aimed to explore whether paediatric neurosurgeons had experience of VP shunt malfunction in relation to rugby participation and, if so to identify the type of shunt problem. We also assessed the level of rugby participation that paediatric neurosurgeons would consider appropriate, including whether they would advocate the use of a scrum cap. In addition, a review of the existing literature was undertaken to identify current evidence and consensus regarding VP shunts and participation in rugby and other contact sports.

## Results

A total of 70 members of the BPNG were identified and asked to complete the survey. An overall response rate of 42.8% (30 out of 70 members) was achieved.

### Question 1

Of the respondents, only one, 3% (1/30), reported to having witnessed a shunt issue directly related to rugby, with the mechanism described as shunt disconnection.

### Question 2

All respondents 100% (30/30) responded yes, that they would allow a 7-year-old child with a VP shunt to play touch rugby.

### Question 3

80% (24/30) of respondents stated, yes, that they would allow a 14-year-old with a VP shunt to play contact rugby.

### Question 4

77% (23/30) of respondents stated, yes, that they would allow an 18-year-old with a VP shunt to play professional/elite rugby.

### Question 5

**Fig. 1:**
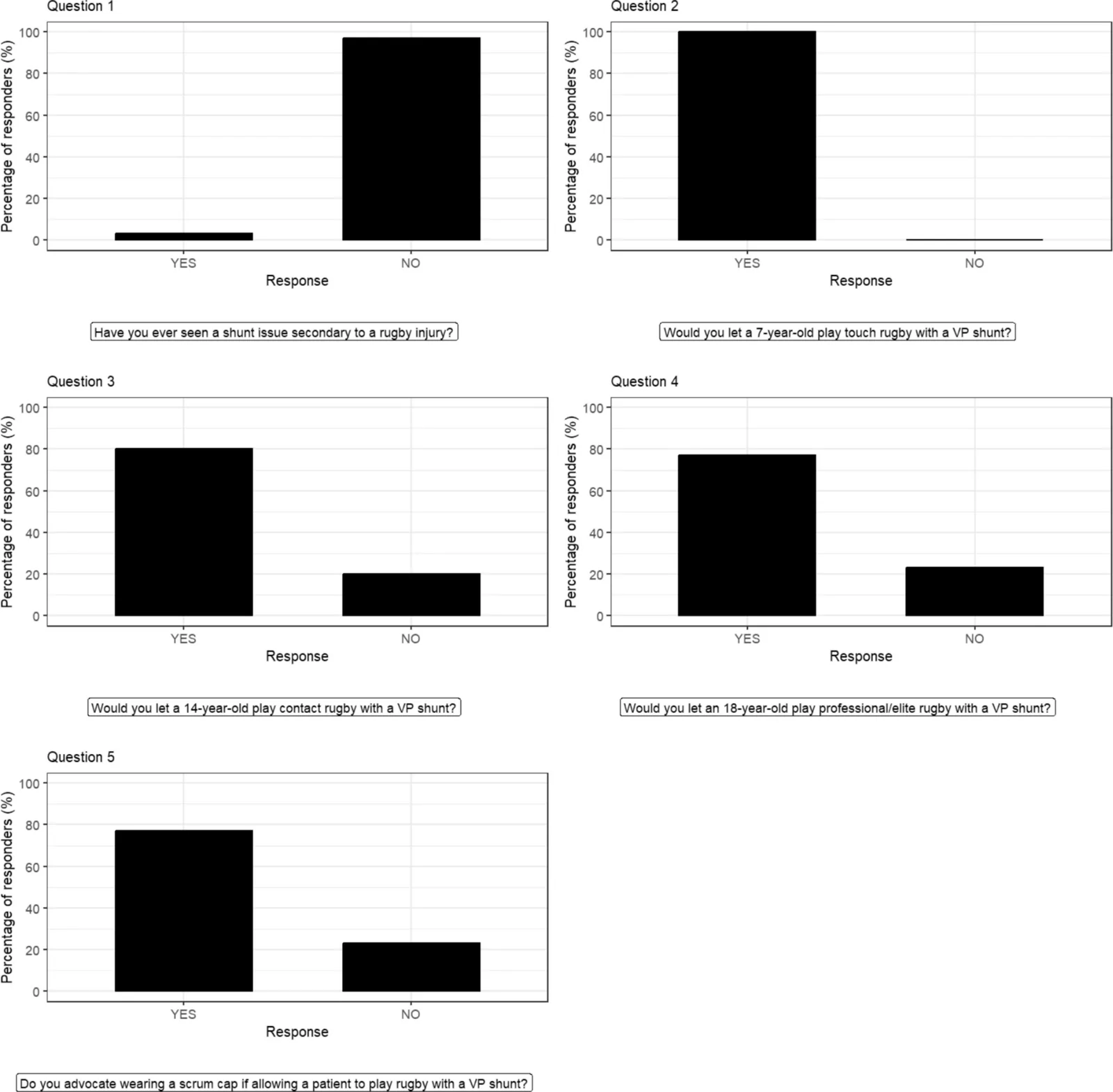
Response to each of the 5 questions

77% (23/30) respondents answered yes, whilst 23% (7/30) answered no to advocating wearing a scrum cap to play (see Fig. 1).

## Discussion and Clinical Implications 

Our study suggests that most paediatric neurosurgeons would allow children with VP shunts to participate in rugby, including at a high level, reflecting current clinical practice. One respondent reported a rugby related shunt complication, specifically disconnection; however, as the timing was unclear and imaging may have been prompted by the sporting event, a causal relationship cannot definitively be established. All respondents (100%) would permit a 7-year-old with a VP shunt to play touch rugby, while 20% would restrict a 14-year-old to play contact rugby and 23% would advise against an 18-year-old participating in professional rugby, with 77% overall supporting return to rugby. Drawing on the experience of BPNG members, this survey reflects insights from hundreds of years of paediatric neurosurgical practice. Most respondents (77%) would recommend the use of scrum caps on return to play, while the remaining 23%, who did not recommend protective headgear, corresponded to those who would not advise return to rugby, suggesting that headgear recommendation was primarily considered by those who supported return to play.

Our findings align with existing literature. One study of 110 children with VP shunts, showed of the 73% who participated in school physical education only 5% reported sport related injuries and no shunt related injuries were reported, supporting the safety of non-collision sports [3]. Evidence for contact sports is limited but largely reassuring, an internet-based survey of paediatric neurosurgeons calculated from the data collected the incidence of sport related shunt complications in children is < 1% [4]. Opinions on contact sports varied, approximately one third imposed no restriction, one third restrict ‘high risk activities’, and one third advise against contact sport – despite the literature indicating minimal risk. Among an estimated 125,000 individuals with VP shunts in the US, no cases of sports related shunt injury have been documented in the medical legal literature, and the incidence of complications in a survey conducted was reported to be < 1% [5]. Despite strong evidence supporting the safety of contact sports, a consistent finding in the literature is approximately one third of paediatric neurosurgeons remain cautious and restrict participation. In the absence of formal guidelines or consensus, decisions are largely judgement based, with a study finding that over 90% of neurosurgeons reporting reliance on personal experience [6]. Reluctance could be due to medical legal concerns, the perception of rare but serious complications, and the paucity of large sport specific studies [5]. These findings do not apply to patients with VP shunts, who then encounter comorbidities such as epilepsy, neurodegenerative diseases and raised intracranial pressure [4, 5, 7, 8].

A consistent finding in the literature and our study is that parental concern may limit children’s engagement in sports. In the study of children with shunted hydrocephalus, exclusion from physical education was mostly driven by parental fear rather than medical contraindications. One respondent to our survey noted, “most parents are not disappointed by me vetoing contact rugby at school”. This highlights while paediatric neurosurgeons provide expert guidance, the ultimate decision rests with the patients and their parents. Advocacy for protective headgear remains important, as even small additional safety measures can make a difference.

## Conclusion

Overall, this survey demonstrates that the majority of British Paediatric neurosurgeons would permit children with ventriculoperitoneal shunts to return to playing rugby, commonly recommending the use of a scrum cap as a precautionary measure to reduce the already low risk of shunt related complications. Despite a relatively small sample size, it is likely that our sample is broadly representative of national practice. These findings are consistent with the existing literature, and reflect the prevailing consensus among paediatric neurosurgeons, which reports a very low incidence of shunt related injury associated with contact sports. Given the well-recognised importance of physical activity and sport in childhood, the ability to support return to play decisions for children with VP shunts is essential. Decisions regarding participation should remain individualised, considering the child’s overall neurological status, parental concerns and the presence of comorbidities.

## Data Availability

No datasets were generated or analysed during the current study.

## Appendix

1. Have you ever seen a shunt issue secondary to a rugby injury? If yes, what was the shunt problem?
2. Would you let a 7-year-old play touch rugby with a VP shunt?
3. Would you let a 14-year-old contact rugby with a VP shunt?
4. Would you let an 18-year-old play professional/elite rugby with a VP shunt?
5. Do you advocate wearing a scrum cap if allowing a patient to play rugby with a VP shunt?
